# Comprehensive well-being scale: development and validation among Chinese in recovery of mental illness in Hong Kong

**DOI:** 10.1186/s40359-021-00686-4

**Published:** 2021-11-13

**Authors:** Will W. L. Sham, Gladys T. Y. Yeung, Winnie W. S. Mak, Candice L. Y. M. Powell

**Affiliations:** 1 New Life Psychiatric Rehabilitation Association, 332 Nam Cheong Street, Kowloon, Hong Kong China; 2 Department of Psychology, The Chinese University of Hong Kong, Shatin, New Territories, Hong Kong China

**Keywords:** Well-being, Personal recovery, Scale development, Validity, Hong Kong, Chinese

## Abstract

**Background:**

Given the absence of a brief scale that reconciles and encompasses different conceptual definitions of well-being (physical, psychological, social and spiritual), the present research aimed at developing and validating a Comprehensive Well-Being Scale (CWBS) that encompasses these different conceptual definition and extend the definition of well-being to transcendental well-being among individuals in recovery of mental illness. The present research focuses on testing the scale among people in recovery of mental illness so that a brief and theoretically comprehensive scale would be available for mental health organization to evaluate the well-being of service users, and to develop and evaluate well-being related services.

**Methods:**

A 56-item preliminary well-being scale was developed by a professional panel. In Study 1, 300 mental health service users in Hong Kong were recruited. Twenty items were selected through principal component analysis to form the CWBS. In Study 2, another sample of 300 service users was recruited. Confirmatory factor analysis was done to confirm a two-factor structure. Validity of the scale was also examined.

**Results:**

The CWBS yielded good internal consistency (Cronbach’s alphas = .79–.91). The finding supported a two-factor structure, namely Intrapersonal Well-Being, and Transpersonal Well-Being, *χ*^2^ (169) = 335.61, *p* < .001, CFI = .90, RMSEA = .06, SRMR = .06.

**Conclusions:**

The CWBS established concurrent and construct validity in assessing well-being among Chinese in recovery of mental illness in Hong Kong. It provided theoretical and practical implications for measuring well-being. Theoretically, it extended the concept of well-being to encompass transcendental well-being in model of recovery among individuals recovery from mental illness. Practically, it provided a tool for evaluation of well-being and service development in mental health organization.

## Background

Not long ago, mental health recovery used to mean the amelioration of symptoms. With their focus on symptomatology, psychiatrists and clinical psychologists developed elaborate assessment tools to measure symptom severity levels as well as functioning. The late 1980s saw the recovery movement, an initiative by mental health service users to incorporate a broader concept of recovery. William Anthony [1] then laid out the vision of personal recovery in mental health services. Today, personal recovery is defined by the Substance Abuse and Mental Health Services Administration (SAMHSA) [2] of the United States Department of Health and Human Services as a process of change, through which individuals improve their health and wellness, live a self-directed life, and strive to reach their full potential. It is not synonymous with cure [3]. Mental health recovery is holistic, which involves not only the recovery of one’s mental state, but also an individual’s full life spectrum of mind, body, spirit, and community [2]. Along with the development, Keyes [4] proposed a complete state model of mental health, by which mental health and mental illness represent two distinct domains, i.e., the absence of mental illness does not imply the presence of mental health, and the absence of mental health does not imply the presence of mental illness. This suggests that regardless of the presence of mental illness, individuals can flourish and enjoy well-being at multiple domains. For example, personal recovery of people with schizophrenia was found to positively predict well-being above and beyond clinical and functional recovery [5]. Adopting this model, Provencher and Keyes [6] augmented the promotion of personal recovery to include positive mental health as an additional outcome. As seen from the latest development in mental health recovery, well-being is an important element that has to be incorporated in assessment, service planning and evaluation in mental health organization. The following sections will outline current definitions of well-being, highlight the importance of the impact of spiritual well-being to mental health and our proposal to develop a comprehensive well-being measure for people in recovery of mental illness.

### Definitions of well-being

Well-being has long been defined by the World Health Organization (WHO) [7] as being multi-faceted, composing of physical, mental, and social dimensions beyond the absence of disease. In particular, mental well-being is further defined as the abilities of individuals to cope with daily stressors, contribute productively in the community, and actualize their potentials [8]. These components of mental well-being have been categorized as hedonic well-being, eudaimonic well-being, and social well-being [9–12]. Hedonic well-being, also known as emotional well-being or subjective well-being, involves life satisfaction, presence of positive affect, and absence of negative affect [10, 13]. This narrow rendition of happiness was deemed inadequate [14], which gave rise to the other concept of eudaimonic well-being. Deci and Ryan [15] highlighted three basic psychological needs, namely autonomy, competence and relatedness, as a core of eudaimonic well-being. Seligman’s PERMA model [16] focuses on five elements of eudaimonic well-being: positive emotions, engagement, meaning, positive relationships, and accomplishment. Ryff [14, 17] derived six elements of eudaimonic well-being, namely self-acceptance, purpose in life, autonomy, positive relations with others, environmental mastery, and personal growth. Furthermore, Keyes [9] extended eudaimonic well-being from Ryff’s [14] intrapersonal model to an interpersonal focus, and came up with five elements of social well-being, namely social coherence, social acceptance, social actualization, social contribution, and social integration. Put together, intrapersonal and interpersonal well-being are believed to be in congruence with the WHO’s definition of mental health, as well as a comprehensive working definition of well-being.

Despite its multidimensionality, the WHO’s definition of mental health has been criticized for missing a fourth dimension—spiritual well-being [18–21]. The body-mind-spirit model has had a long history in Western religions like Christianity [22] as well as Eastern religions and philosophies including Confucianism, Taoism, Buddhism, and Hinduism [23]. Nevertheless, spirituality, religion, and personal beliefs are not synonymous [24]. One of the earliest definitions of spiritual well-being by the National Interfaith Coalition on Aging (NICA) [25] is the affirmation of life in the relationship with oneself, others, nature, and God. Following the NICA definition, Fisher [26] proposed the spiritual well-being model, pertaining to domains of personal, communal, environmental, and transcendental well-being. According to Fisher [26], personal domain deals with how one intrarelates with oneself with regard to meaning, purpose, and values in life; communal domain is expressed in the quality and depth of interpersonal relationships, between the self and others, relating to morality, social justice, culture, and religion; environmental domain includes the care and nurture for the physical environment and other organisms, as well as a sense of awe and wonder; transcendental domain is the relationship of the self with something or someone beyond humanity (i.e., ultimate concern, cosmic force, transcendent reality, or God).

### Importance of spiritual well-being to mental health

Spiritual well-being is important to mental health from the perspective of people in recovery [27]. In particular, research suggested that spiritual well-being is related to reduced anxiety [28, 29], depressive symptoms [28, 29], suicidal ideation [28] and improved post-traumatic recovery [29]. Despite the importance of spiritual and transcendental well-being in the mental health recovery, this aspect of well-being was not emphasized in models of mental health recovery. For instance, in the 10 guiding principles of recovery proposed by SAMHSA [2], spirituality was only very briefly mentioned under the guiding principle that recovery is holistic: ‘recovery encompasses an individual’s whole life, including mind, body, spirit, and community’. It was also not clear whether the spirit and community component encompasses aspects of communal and transcendental well-being, which highlights how an individual relates with people and things beyond themselves, including people in the community as well as the environment and the Divine [26]. Furthermore, the well-being model proposed by Keyes [9] extended the definition of well-being to a social one, but dimensions of spiritual well-being are not covered. Thus, we proposed that a comprehensive well-being model for individuals in recovery of mental illness should include physical, psychological, social and spiritual well-being.

### Intrapersonal and transpersonal well-being

In this research, we proposed domains of Intrapersonal and Transpersonal Well-being to reconcile the overlaps between different conceptual definitions of well-being, as well as to fill in the existing research gap and extend the concept of well-being in people in recovery to a more comprehensive definition. As reviewed in previous sections, the personal domain of spiritual well-being proposed by Fisher [26] and the psychological well-being proposed by Ryff [14] both concern how an individual intrarelates with themselves. Also, the communal domain of spiritual well-being proposed by Fisher [26] and the social well-being proposed by Keyes [9] both regard how an individual interrelates with other people. Furthermore, Fisher’s [26] other domains of spiritual well-being, i.e., environmental and transcendental well-being, extend beyond how an individual interrelates with other people to how an individual interrelates with the environment and something or someone beyond humanity. To cover the above aspects of well-being, we proposed the following operational definitions of well-being: intrapersonal well-being as involving how an individual intrarelates with themselves, including positive physical health and a positive sense of the self [13, 14]; transpersonal well-being as encompassing how an individual relates with people and things beyond themselves, including people in the community [9] as well as the environment and the Divine [26].

### Purpose of scale development

To the best of our knowledge, no scale has been developed to encompass all the domains of well-being that have been discussed in literature. A recent systematic review of 99 well-being scales identified as many as 196 dimensions that clustered around six key themes, while pinpointing the ambiguity around the conceptual similarities and differences among different dimensions [30]. Existing scales measuring well-being mainly encompass physical, psychological and social well-being, for instance, Ryff Scales of Psychological Well-being [14, 50], and Mental Health Continuum Short Form [4], but do not extend to domains of spiritual and transcendental well-being. Existing scales for measuring recovery, for instance, Recovery Assessment Scale [31], Mental Health Recovery Measure [32], and Recovery Self-Assessment [33], are limited to the mind, body, and community, but do not extend to environmental and transcendental well-being.Some scales attempt to encompass more dimensions of well-being, for instance, Body-Mind-Spirit Well-Being Inventory [34] and Holistic Well-Being Scale [35], but do not cover well-being in terms of one’s relationship with the environment and something or someone beyond humanity.

The aims of the present study were to reconcile and extend different conceptual definitions of well-being among people in recovery of mental illness to cover transcendental aspect of well-being, and to develop a comprehensive well-being scale that grasps every intra- and trans-personal domain. The current paper also aimed to provide a practical and efficient tool for mental health organization to routinely measure comprehensive well-being, and to develop and evaluate services that aim to improve the well-being of people in recovery of mental illness. In addition, the scale would also provide mental health service users a brief scale for easier assessment of well-being for increasing awareness and for monitoring of one’s mental health.

In this research, Study 1 aimed at establishing a preliminary well-being scale. This preliminary scale would then be used to form our Comprehensive Well-being Scale using principal component analysis. Study 2 aimed at confirming the factor structure established in Study 1 using confirmatory factor analysis and examining the scale’s concurrent, convergent and discriminant validity.

### Study 1

The aims of Study 1 were to explore the factor structure of the scale and to select items from a preliminary well-being scale through principal component analysis to form a comprehensive well-being scale.

## Method

### Development of a preliminary well-being scale

To develop a preliminary well-being scale, Furr’s guidelines on scale construction [36] were followed. The guideline suggested four steps in scale construction: (1) articulation of construct and context; (2) choosing a response format and assembling initial item pool; (3) collecting data from respondents; and (4) examination of psychometric properties and quality. While step one and two would be described in this section, step three would be described in the method section and step four would be described in the results section.

First, the construct of well-being was articulated through the review of existing well-being literature as described above. We aimed at developing a well-being scale that measures intrapersonal and transpersonal well-being. As for consideration of context of scale development, the development of the scale would consider that the target population is people in recovery of mental illness. In particular, the comprehensibility and length of the scale would be considered in the process of scale development and would be described in greater details in the parts below. Second, following step two of Furr’s guideline [36], a pool of 110 items were adopted with modification and reformulated into Chinese from existing measures that tap into the two domains of well-being. Intrapersonal well-being measures included Rosenberg Self-esteem Scale [37], Ryff Scales of Psychological Well-being [12, 51], State Hope Scale [38], Emotion Reactivity Scale [39], and Nonattachment Scale [40]; four new items were proposed by a professional panel to cover the aspect of physical health. Transpersonal well-being measures included Social Connectedness Scale [41], Active and Engaged Citizenship [42], and Humanitarianism-Egalitarianism Scale [43]; three new items were proposed by the professional panel to cover the aspect of common humanity. Other measures that tap into several domains of well-being were also included: Five Facet Mindfulness Questionnaire [44], Flourishing Scale [45], and Mindful Flourishing Scale (Mak & Chow, unpublished scale).

The pool of 110 items adopted was proposed for professional panel discussion. Panelists included two clinical psychologists, one post-doctoral fellow with background in social work and psychology and one psychological well-being officer experienced in mental health and personal recovery. The panel voted on the items basing on the extent to which they measure intrapersonal and transpersonal well-being. The voting determined whether the item was initially considered as measuring intrapersonal and transpersonal well-being. Next, items that appeared to have similar wordings were grouped together. For example, the item “When I look at the story of my life, I am pleased with how things have turned out” from the Ryff Scales of Psychological Well-being [14, 50] and the item “On the whole, I am satisfied with myself” from the Rosenberg Self-esteem Scale [37] were rephrased into a single item “I feel satisfied with my life.” After discussion, a 56-item preliminary scale was drafted.

As the scale was intended to be used to assess the well-being of people in recovery of mental illness, comments from people in recovery were sought on the comprehensibility of the preliminary well-being items and on suitable length of the scale in terms of how long they can sustain their attention when completing the items. Wordings were revised so that people in recovery who helped comment on the preliminary scale can easily understand. Also, as the scale would be used primarily by mental health organizations, likely together with other scales, the scale’s brevity was our main focus. Basing on people in recovery’s comment on suitable length and the expert panel’s experience in administration of scales to people in recovery, we aimed at creating a one-page brief scale with around 20 items.

### Participants and procedures

Convenience sampling was used in the study. Participants were recruited from the largest non-governmental organization providing comprehensive community-based mental health services in Hong Kong. They are individuals with mental illness currently receiving mental health service. We made a list of all service users from each of the 30 service units in the organization, excluding those who had intellectual disability or dementia, did not understand Cantonese or Chinese, or refused to participate in the study. For each list, a number was randomly assigned to each eligible service user. They were then arranged in ascending order of the randomized numbers. The number of participants invited from each list was proportional to the number of service users from each unit, making the composition of the participants a representative sample of the distribution of all service users in different centers. As a rule of thumb, a sample size of 300 is considered good for factor analyses [46]. Therefore, a total of 300 service users (132 males and 168 females) were recruited in the present study, with a mean age of 49.25 (*SD* = 12.25, range = 19–77). Details on marital status, education level, and occupation could be found in Table 1.

**Table 1:** Demographics of participants in Study 1 and Study 2

	Study 1 (%)	Study 2 (%)
Marital status		
Single	49.7	50.0
Married	22.8	23.2
Divorced/separated	27.4	26.2
Education level		
Primary	23.3	20.5
Secondary	68.0	65.5
Tertiary	8.7	14.1
Occupation		
Full-time	28.1	26.6
Part-time	11.5	11.1
Student	0.3	2.0
Home-maker	15.6	18.5
Unemployed	26.4	15.5
Retired	15.9	15.2

Data collection was conducted in a group format with 3 to 6 service users. Informed consent forms were completed. Participants were then given a set of questionnaires for self-administration. A student helper was available in each data collection session to assist participants with filling in the questionnaires. All participants were given HKD50 (~ USD6) supermarket coupon as incentives. The study was approved by the Survey and Behavioural Research Ethics Committee, the Chinese University of Hong Kong.

### Measures

The 56-item preliminary well-being scale was administered. Participants rated the items based on a 5-point Likert scale from 1 (strongly disagree) to 5 (strongly agree).

### Analysis

To examine the factor structure of the preliminary well-being scale, principal component analysis was conducted on the correlation matrix of the 56 self-developed items. The Kaiser–Meyer–Olkin (KMO) test [47] and Bartlett’s [48] test of sphericity were used to measure the sampling adequacy for conducting exploratory factor analysis. A KMO index greater than 0.6 and a significant result from the Bartlett’s test of sphericity suggest suitability [49].

## Results

KMO value was 0.93, while Bartlett’s test of sphericity was found significant, *χ*^2^ (1540) = 9535.40, *p* < 0.001, suggesting sufficient item dependency among these 56 items for conducting exploratory factor analysis.

In terms of factor extraction, we relied on the results from Cattell’s Scree Test as well as the interpretability of the factors. Overall speaking, a two-factor structure on the well-being was suggested, accounting for 39.7% of variance of the data. Based on the oblique rotation, a total of 34 items was loaded on the first factor with non-trivial factor loadings (i.e., *λ* > 0.40), while 17 items were loaded on the second factor (Table 2). Five items were dropped since they were weakly associated with both two factors. As a result, 51 items were included under a two-factor model. The first factor accounted for 33.7% of variance among items (eigenvalue = 18.87); the items loaded on this factor are about how an individual intrarelates with themselves and was labelled as “Intrapersonal Well-Being.” The second factor accounted for 6.0% of variance among items (eigenvalue = 3.37); the items loaded on the second factor are about how an individual relates with people and things beyond themselves and was labelled as “Transpersonal Well-Being.”

**Table 2:** Factor loadings of the items in Study 1

Items		Factor 1	Factor 2
Q36	I feel happy	**.821**	−.061
Q47	I have a healthy body	**.817**	−.112
Q51	I feel full of energy	**.816**	−.073
Q50	I feel satisfied with my life	**.801**	−.069
Q37	I feel sprightly	**.798**	−.070
Q17	I can focus on what I am doing	**.780**	−.092
Q40	I feel calm	**.731**	−.069
Q41	I can manage my everyday life with my physical strength	**.714**	−.058
Q45	I can live with life’s ups and downs and let go of unpleasant past experience	**.709**	.022
Q30	When something bad happens, I can adjust my emotions	**.692**	.031
Q34	I have a strong body	**.692**	−.054
Q16	I get enough sleep	**.686**	−.203
Q8	I am good at taking care of my physical health	**.684**	.048
Q46	I feel blissful and warm	**.680**	.078
Q29	I am aware of my emotions and won’t be trapped in them	**.675**	.006
Q15	I have a life goal	**.667**	.065
Q25	I see my value	**.661**	.134
Q18	I am aware of my body and health status	**.636**	−.078
Q11	I treat myself with kindness	**.632**	−.019
Q3	I am leading a meaningful life	**.626**	.075
Q14	I am having a healthy diet	**.612**	.039
Q21	I accept new challenges	**.599**	.085
Q27	In the face of difficulties, I insist on actively finding ways to solve problems	**.597**	.092
Q54	I am having a satisfying social life	**.589**	.155
Q5	I can clearly express my emotions	**.571**	.010
Q22	I know my values well	**.560**	.127
Q4	I practice ways to maintain good health	**.530**	.145
Q31	I feel comfortable with my weaknesses	**.528**	−.002
Q20	I participate in activities that need interactions with people	**.496**	.170
Q1	I am exercising enough	**.445**	.109
Q19	I am aware of my mood swings	**.429**	.064
Q10	I am learning new stuff	**.429**	.192
Q32	I appreciate my strengths	**.427**	.193
Q53	I can reflect on my past mistakes and failures with an open mind	**.421**	.126
Q56	I have family and friends who trust and support me	.352	.329
Q49	I am connected with people around me (e.g., friends, family, partner)	.329	.300
Q43	When I hear stories of a stranger, I can feel connected with them	−.111	**.714**
Q42	When I hear bad things happened to a stranger, I would feel their pain	−.249	**.708**
Q52	The environment and I are closely connected (e.g., discrimination, environmental quality, social atmosphere, etc.)	−.061	**.639**
Q13	Given the chance, I would participate in activities to promote social changes	.058	**.586**
Q33	I treat others with kindness	.118	**.562**
Q38	I think everyone, however different in gender, age, color or class, should have equal opportunities and voice	−.095	**.551**
Q55	I would feel happy for the happiness of people around me	.129	**.545**
Q26	I am grateful to people around me for all they have done to me	.118	**.525**
Q23	I care about what is happening in society	.201	**.517**
Q39	I feel like part of the whole society	.243	**.489**
Q48	I am aware of the impact of the environment on me (e.g., social atmosphere, environmental quality, discrimination, etc.)	−.081	**.474**
Q44	I have a sense of belonging to the community or society in which I live	.197	**.458**
Q12	I would participate in activities to promote social changes (e.g., environmental protection, air pollution, social poverty, sexual orientation discrimination, etc.)	.287	**.450**
Q28	I can accept the inadequacies of others	.200	**.422**
Q24	I am connected with the nature (e.g., appreciating or protecting the nature)	.264	**.417**
Q35	Even for strangers, I can also see my similarities with them	.266	**.409**
Q9	I have noticed what is happening around the world	.296	**.404**
Q6	I would participate in activities that cultivate spirituality (e.g., going to church, meditation, devotion, praying, mindfulness, practicing Confucianism, Buddhism and Taoism)	.085	.381
Q7	I pay attention to health information (e.g., reading newspapers/magazines, watching television shows, listening to radio programs and attending health talks in order to obtain different health information)	.231	.381
Q2	I feel that social current affairs and I are closely related	.130	.333

Bolded values indicate that items are loaded on the specific factor in the bolded column

Factor 1 = Intrapersonal Well-Being; Factor 2 = Transpersonal Well-Being

To develop a brief scale with equal number of items measuring both Intrapersonal Well-being and Transpersonal Well-being, we selected 10 items with the highest factor loadings from each factor. The factor loading also has to be greater than 0.4 to be considered acceptable [46].The factor loadings of 10 items from the factor of Intrapersonal Well-Being were all significant, ranging from 0.69 to 0.82, while the factor loadings of the 10 items from the factor of Transpersonal Well-Being were also significant, ranging from 0.49 to 0.71. The Cronbach’s alphas for Intrapersonal Well-Being and Transpersonal Well-Being were 0.93 and 0.83, respectively.

### Study 2

The aim of Study 2 was to confirm the factor structure of the CWBS using confirmatory factor analysis and to establish the concurrent, convergent and discriminant validity of the scale with reference to other existing well-being scales.

Concurrent validity of the scale was examined by correlating CWBS to several other well-being scales that encompass different domains of well-being. Given that recovery is theoretically a result of good well-being e.g. [2, 4, 6], concurrent validity with an assessment of recovery was also examined. Both factors of Intrapersonal Well-Being and Transpersonal Well-Being were hypothesized to be positively and moderately associated with measures of well-being and recovery.

Construct validity of CWBS was examined using existing measures that tap into different domains of well-being. Given our operational definitions as discussed above, Intrapersonal Well-being was hypothesized to be negatively and moderately associated with physical symptoms, depressive symptoms, anxiety symptoms, and psychological distress; Transpersonal Well-being was hypothesized to be positively and moderately associated with compassionate love, social connectedness, universalism, and self-transcendence, and negatively and moderately associated with alienation. These measures were used as construct validity indicators because most of them had been validated among Chinese, and have strong theoretical relevance to their respective domain of well-being e.g. [4, 14, 26].

## Method

### Participants and procedures

Participant recruitment and data collection procedure were the same as Study 1. Another group of 300 service users (118 males and 182 females) were recruited through a similar process as in Study 1, with a mean age of 49.54 (*SD* = 12.46, range = 19–74). Participants of Study 1 were excluded in this study. Details on marital status, education level, and occupation could be found in Table 1.

### Measures

The CWBS developed in Study 1 was used. It contains 20 items and measures two domains of well-being, namely Intrapersonal Well-Being and Transpersonal Well-Being. The following scales were used to examine validity of the CWBS.

Mental Health Continuum Short Form [4]. It is a 14-item scale measuring emotional, psychological and social well-being. It was translated into Chinese and independently back-translated into English for checking its accuracy.

Holistic Well-Being Scale [35]. The Holistic Well-being Scale contains 30-items and measures affliction (emotional vulnerability, bodily irritability and spiritual disorientation) and equanimity (non-attachment, mindful awareness, general vitality and spiritual self-care). The scale is in Chinese.

Ryff Scales of Psychological Well-being [14, 50]. It is an 18-item scale measuring six aspects of psychological well-being, including self-acceptance, positive relations with others, autonomy, environmental mastery, purpose in life and personal growth. The scale was translated into Chinese and independently back-translated into English for checking its accuracy.

Recovery Assessment Scale [51]. The Recovery Assessment Scale is a 24-item scale measuring recovery in terms of personal confidence and hope, willingness to ask for help, goal and success orientation, reliance on others, and no domination by symptoms. The validated Chinese version [52] was used in the present study.

Body-Mind-Spirit Well-Being Inventory [35]. Physical symptoms was measured by the 14-item Physical Distress sub-scale of the Body-Mind-Spirit Well-Being Inventory [35]. It measures the level of subjective physical distress caused by specific physical symptoms such as “headache,” “dizziness” and “palpitation.” The scale is in Chinese.

Patient Health Questionnaire-9 [53]. Depressive symptoms were measured by the 9-item Patient Health Questionnaire 9 scale [53] The Chinese version was validated [54, 55] and used in the present study.

Generalized Anxiety Disorder-7 [56]. Anxiety symptoms were measured by the Generalized Anxiety Disorder 7-item scale [56]. The Chinese version of the scale was validated [57] and used in the present study.

Kessler Psychological Distress Scale (K6) [58]. It is a 6-item scale that measures psychological distress. The Cantonese-Chinese version of the scale was translated and validated by Lee and colleagues [59] and used in the present study.

Santa Clara Brief Compassion Scale [60]. It is a 5-item scale that measures compassionate love. It was developed from Sprecher and Fehr’s Compassionate Love Scale [61]. The scale was translated into Chinese and independently back-translated into English for checking its accuracy.

Social Connectedness Scale [41]. It is a 8-item scale measuring social connectedness. All items were reverse-coded, so that a higher score indicates a higher level of social connectedness. The scale was translated into Chinese and independently back-translated into English for checking its accuracy.

Portrait Values Questionnaire 5X Value Survey [62]. Universalism was measured by the universalism-concern and universalism-tolerance subscales of the Portrait Values Questionnaire 5X Value Survey [62]. They measure the concern and tolerance for the welfare of all people and nature. The pronoun was changed from “he/him” to “I/me.” A total score for universalism was calculated by summing up scores from the two subscales. The scale was translated into Chinese and independently back-translated into English for checking its accuracy.

Adult Self-Transcendence Inventory [63]. It is an 18-item scale that measures self-transcendence and alienation. The inventory consisted of two subscales, namely self-transcendence and alienation. Self-transcendence reflects a decreasing reliance on externals for definition of the self, increasing interiority and spirituality, and a greater sense of connectedness with past and future generations. Alienation measures the negative affect as a result of social isolation [63]. The scale was translated into Chinese and independently back-translated into English for checking its accuracy.

### Analysis

Confirmatory factor analysis (CFA) was conducted to examine internal consistency and factorial validity of the CWBS. According to the criterion by Nunnally [64], a Cronbach’s alpha higher than 0.70 is considered good internal consistency. Assessment of model fit was based on multiple criteria, including absolute misfit and incremental fit indices. A CFA model with Root-Mean-Square Errors of Approximation (RMSEA) [65] < 0.08, Standardized Root Mean Squared Residual (SRMR) [66] < 0.08 and Comparative Fit Index (CFI) [67] > 0.90 would be regarded as yielding acceptable fit to the data [68]. In addition, Cohen’s [69] guideline was used to determine the strengths of correlation coefficients, with *r* around 0.1 indicating small effect sizes, *r* around 0.3 indicating medium effect sizes, and *r* > 0.5 indicating large effect sizes.

Concurrent, convergent and discriminant validity are examined by correlating Intrapersonal Well-Being and Transpersonal Well-being factors of the CWBS to different measures of physical, psychological and spiritual well-being.

## Results

### Internal consistency

The Cronbach’s alphas of Intrapersonal Well-Being and Transpersonal Well-Being were 0.91 and 0.79, respectively. Taken as a whole, these findings suggested good internal consistency.

### Factorial validity

Based on the 20 items with the highest factor loadings found in a two factor-structure in Study 1, we attempted to cross-validate the factor structure of the CWBS using CFA.

**Fig. 1:**
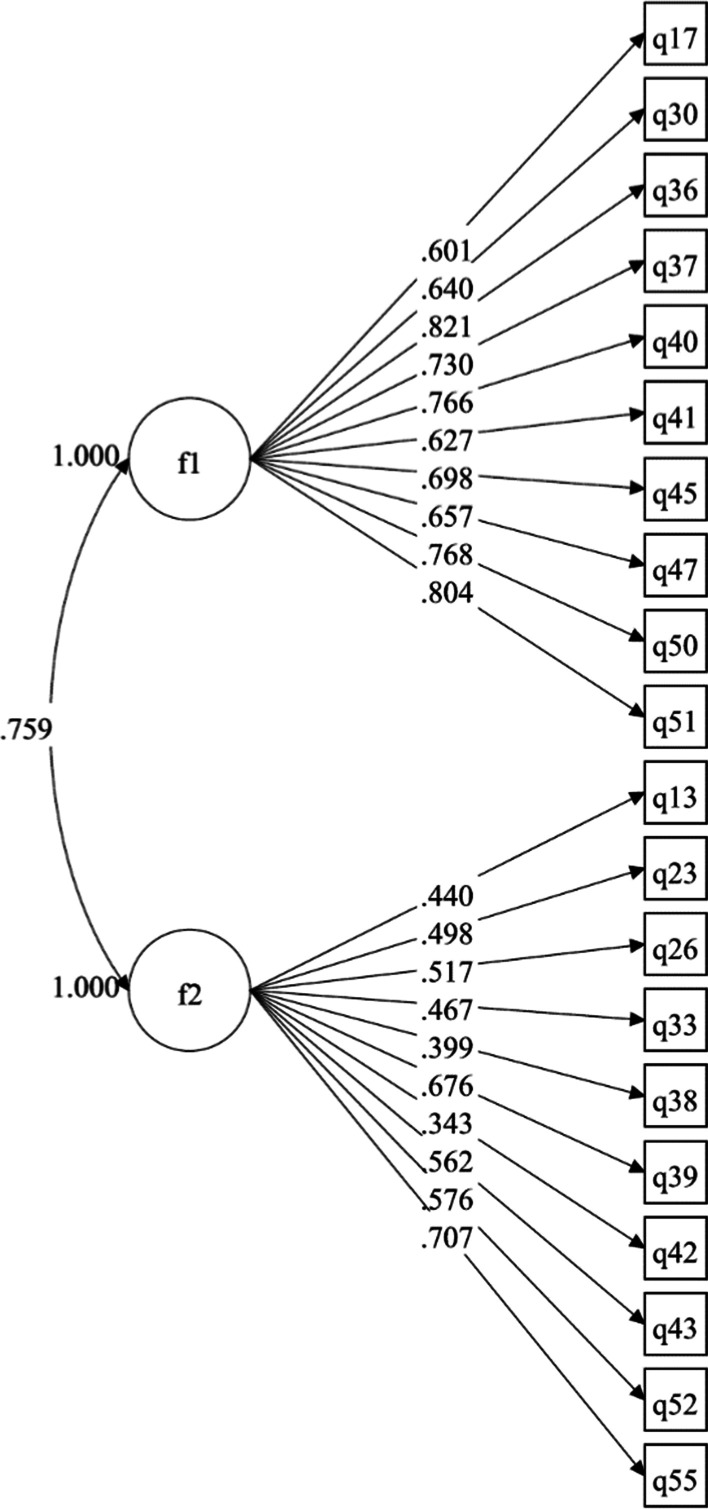
The two-factor confirmatory factor analysis model in Study 2

Overall speaking, a two-factor CFA model yielded acceptable fit to the data, *χ*^2^ (169) = 335.61, *p* < 0.001, CFI = 0.90, RMSEA = 0.06, SRMR = 0.06, confirming the factorial validity of the well-being. In the CFA model (Fig. 1), items had moderate to high factor loadings in both factors. For Intrapersonal Well-Being, factors loadings were all significant and ranged from 0.60 to 0.82, with an average of 0.71. For Transpersonal Well-Being, all factors loadings were also significant and ranged from 0.34 to 0.71, with an average of 0.52. In the CFA model, the two latent factors were significantly and positively correlated, *r* = 0.76, *p* < 0.001.

### Concurrent validity

Concurrent validity of the CWBS was conducted by investigating the correlations between its two factors and well-being and recovery. As shown in Table 3, both factors showed positive and large correlations with emotional, psychological and social well-being, holistic well-being, psychological well-being, as well as recovery.

**Table 3:** Correlations of the two factors of the CWBS with different measures

	Intrapersonal Well-Being	Transpersonal Well-Being
1. Emotional, psychological and social well-being	.71***	.59***
2. Holistic well-being	.72***	.56***
3. Psychological well-being	.58***	.56***
4. Recovery	.66***	.60***
5. Physical symptoms	−.43***	−.21***
6. Depressive symptoms	−.54***	−.34***
7. Anxiety symptoms	−.52***	−.31***
8. Psychological distress	−.63***	−.40***
9. Compassionate love	.19*	.45***
10. Social connectedness	.37***	.43***
11. Universalism	.23**	.47***
12. Self-transcendence	.54***	.52***
13. Alienation	−.45***	−.35***

^*^p < .05, **p < .01, ***p < .001

### Convergent validity

Convergent validity of the CWBS was conducted by investigating the correlations between its two factors and different measures that tap into different domains of well-being. As shown in Table 3, Intrapersonal Well-being was negatively and strongly associated with depressive symptoms, anxiety symptoms, and psychological distress, and was negatively and moderately associated with physical symptoms. Transpersonal Well-being was positively and strongly associated with self-transcendence, and positively and moderately associated with compassionate love, social connectedness, and universalism. It was negatively and moderately associated with alienation as well.

### Discriminant validity

To further examine discriminant validity of the CWBS, regression analyses were conducted, with both of its factors predicting different measures. As shown in Table 4, after controlling for the other factor, only Intrapersonal Well-being but not Transpersonal Well-being predicted physical symptoms, depressive symptoms, anxiety symptoms, and psychological distress. On the other hand, after controlling for the other factor, only Transpersonal Well-Being but not Intrapersonal Well-being predicted compassionate love, social connectedness, and universalism. Nevertheless, after controlling for the other factor, both factors still predicted self-transcendence and alienation.

**Table 4:** Standardized coefficients of regression analyses with the two factors of the CWBS predicting different measures

	Intrapersonal Well-Being	Transpersonal Well-Being
1. Physical symptoms	−.47***	.08
2. Depressive symptoms	−.53***	−.01
3. Anxiety symptoms	−.53***	.01
4. Psychological distress	−.62***	−.01
5. Compassionate love	−.15	.54***
6. Social connectedness	.16	.33**
7. Universalism	−.10	.52***
8. Self-transcendence	.35***	.31***
9. Alienation	−.37***	−.12^†^

^†^*p* < .01

^*^p < .05

^**^p < .01

^***^p < .001

## Discussions

### Summary of findings

Study 1 aimed at exploring the factor structure of the CWBS and item selection. Study 2 aimed at examining the reliability and validity of the CWBS constructed from Study 1 among a sample of people in recovery in Hong Kong. The CWBS was shown to have sound psychometric properties in measuring well-being.

The two factors of the CWBS yielded satisfactory internal consistency, suggesting that it is a scale with homogeneous items measuring the same underlying construct. A two-factor model was found and confirmed in the CWBS. The two factors—Intrapersonal Well-Being and Transpersonal Well-Being—not only encompass the physical, mental, and social domains of the WHO’s [7] definition of mental health, but also extend to one’s connection with things beyond the self and humanity.

To examine the concurrent validity of the CWBS, we compared it with other existing scales measuring well-being and recovery. Concurrent validity is evident with Ryff’s [14] psychological well-being, Chan and colleagues’ [34] holistic well-being, as well as Keyes’s [4] emotional, psychological and social well-being. In addition to well-being, concurrent validity of the CWBS with recovery was demonstrated. The measuring of well-being not only informs the general population, but also sheds light on people in recovery. This is in line with Keyes’s [4] complete state model of mental health, introducing mental health, in addition to the absence of mental illness, as another distinct domain of recovery.

Construct validity of the CWBS was demonstrated by its convergent and discriminant validity. As hypothesized, Intrapersonal Well-Being was found to be associated with measures that tap into its corresponding constructs, i.e., physical symptoms, depressive symptoms, anxiety symptoms, and psychological distress. Transpersonal Well-Being was also found to be associated with measures that tap into its corresponding constructs, i.e., compassionate love, social connectedness, universalism, self-transcendence, and alienation. After controlling for the other factor, the two factors further demonstrated their discriminant validity, confirming the disassociations with unrelated constructs. The only exception is that after controlling for the other factor, both factors are still associated with self-transcendence and alienation. A possible explanation is that the Adult Self-Transcendence Inventory [63], in addition to measuring self-transcendence and alienation, takes also into account the positive and negative affects resulting from them. In fact, self-transcendence was found to be correlated with emotional well-being [70]. Besides, its strong emphasis on the self might deviate from our definition of Transpersonal Well-Being, which emphasizes more on the connectedness with society, environment, and the world at large.

### Implications

The CWBS has a number of implications. Theoretically, it reconciles the overlaps between different conceptual definitions of well-being within the current literature framework, including Ryff’s [14] psychological well-being, Keyes’s [9] social well-being, and Fisher’s [26] spiritual well-being, and extends the concept to encompass transcendental well-being among individuals in recovery of mental illness. The extension is important in suggesting that current recovery model may need to include aspects of transpersonal well-being in order to have a more comprehensive conceptual and service model. Practically, it provides a comprehensive and easily administrable tool for measuring well-being. It is purported to be used by mental health practitioners and organizations who wish to incorporate a comprehensive measurement of well-being in their service, to develop services that aim to improve the well-being of people in recovery of mental illness, as well as to evaluate such services’ effectiveness. It can also be used by people in recovery of mental illness to monitor their well-being. Upon further validation studies, it can potentially be used by individuals in the general public as a check-up of their well-being status.

### Limitations

Despite solid empirical evidence for the reliability and validity of the CWBS, the present study is not without its limitations. First, it is difficult to strike a balance between a broad and a brief scale, so that it could encompass the multidimensionality of well-being, and at the same time be more manageable to be used as a regular assessment for each service user in mental health organizations. Future studies could refine or even expand the items to explore the possibility of more domains. Second, our sample limits the generalizability of the findings to the general population of Hong Kong. Although the present sample involved service users from several service centers across different regions in Hong Kong, it mainly applies to people in recovery recruited from a single mental health organization. Future validation studies could consider using a more heterogeneous sample, both within and outside the mental health service sector. Third, despite efforts in establishing concurrent and construct validity of the CWBS with other scales that tap into different domains of well-being, some of the scales were translated but yet to be validated in a Chinese population. The validation results should be interpreted with caution. Much research is needed to establish psychometrically sound measures of well-being in general Chinese community. The CWBS may be tested in the general Chinese community in future study and compare the results from general public with current results obtained among people in recovery.

## Conclusions

The present study is the first to expand the measure of well-being from intrapersonal and interpersonal to transpersonal among individuals in recovery of mental illness. Study 1 developed a 20-item CWBS, measuring Intrapersonal Well-Being and Transpersonal Well-Being. Study 2 provided evidence for the CWBS’s reliability, factorial validity, concurrent validity, convergent validity, and discriminant validity among Chinese in recovery of mental illness in Hong Kong. The CWBS paves the way for researchers to understand different domains of well-being, for mental health service providers to administer a broad yet brief well-being scale, for mental health service users to assess their well-being along their recovery journey, and for the public to easily assess their well-being for human flourishing.

## Data Availability

The datasets generated during the current study are available in the OSF repository, https://osf.io/2hqky/?view_only=196685702d3a412e8c320779224e865e

## Abbreviations

CFA: Confirmatory factor analysis
CFI: Comparative fit index
CWBS: Comprehensive Well-Being Scale
KMO: Kaiser–Meyer–Olkin
NICA: National Interfaith Coalition on Aging
RMSEA: Root-mean-square errors of approximation
SAMHSA: Substance Abuse and Mental Health Services Administration
SRMR: Standardized root mean squared residual
WHO: World Health Organization
